# How the Crosslinker Amount Influences the Final Properties of Hydroxyethyl Methacrylate Cryogels

**DOI:** 10.3390/gels10030163

**Published:** 2024-02-22

**Authors:** Giuseppe Proietto Salanitri, Enrica Luzzi, Daniele Caretti, Tommaso Mecca, Sabrina C. Carroccio, Andrea A. Scamporrino

**Affiliations:** 1 CNR-Institute for Polymers, Composites and Biomaterials, Via Paolo Gaifami 18, 95126 Catania, Italy; sabrinacarola.carroccio@cnr.it (S.C.C.); andreaantonio.scamporrino@cnr.it (A.A.S.); 2 Department of Industrial Chemistry “Toso Montanari”, University of Bologna, Viale Risorgimento 4, 40136 Bologna, Italy; daniele.caretti@unibo.it; 3 Department of Chemical Engineering, Materials and Industrial Production, University of Naples Federico II, DICMaPI–P. le Tecchio 80, 80125 Naples, Italy; enrica.luzzi@unina.it; 4 CNR-ICB, Via Paolo Gaifami 18, 95126 Catania, Italy; tommaso.mecca@icb.cnr.it

**Keywords:** cryogels, crosslinker, HEMA, swelling, adsorption, mechanical properties

## Abstract

The investigation of the mechanical, thermal, and adsorption properties of hydroxyethyl methacrylate (HEMA) cryogels as a function of a reactant ratio is herein reported to better address materials for specific applications. To this aim, cryogels have been synthesized using different monomer/crosslinker (N,N′-methylene-bisacrylamide–MBAA) ratios. The study of SEM images made it possible to identify the trend in the material’s macroporosity. As would be expected, the average measured pore width decreased as the amount of MBAA increased while the number of pores grew. Swelling capacity ranges from 8.7 gW/g_gel_ (grams of water per gram of gel) to 9.3 gW/g_gel_. These values are strictly connected with the pore’s size and distribution, revealing that the water uptake for the most crosslinked sample is inferior to other samples. The equilibrium-adsorption capacity (Qe) towards the methylene violet (MV) was also assessed, revealing no remarkable differences after 24 h of a batch test. As expected, thermogravimetric analysis (TGA) also showed no significant changes in stability that ranged from a maximum weight loss temperature (T Max) of 420 °C to 425 °C, which increased as a function of crosslinker content. Conversely, compression strength measurements showed a notable difference of about 50% in modulus (Ec), moving from the higher to the lower HEMA/MBAA ratio. These new comparative results indicate how slight variations in the reactant’s ratio can steadily improve the mechanical properties of the HEMA cryogel without affecting its adsorption efficiency. This can be helpful in the design of materials for water and energy purposes. Since swelling properties are needed in the case of biomedical applications, the HEMA/MBAA ratio should be tuned versus high values.

## 1. Introduction

Over the past 25 years, the scientific community developed the chemistry of hydrogels to exploit their peculiar features, mainly in biomedical areas such as mimicking soft living tissue and drug release in wound healing [1,2]. This has led to exploring the use of polymer gels in an ever-increasing variety of new products with specific and demanding characteristics. Among them, materials able to satisfy the requests of the different applications area are cryogels. They play a predominant role in environmental and energetic applications [3]. As is well known, their interconnected macroporous structure can confer super absorbance with high and tuneable mechanical properties in hydrophilic and hydrophobic formulations. Cryogels have received remarkable attention in water remediation as adsorbents [4,5], in heterogeneous catalysis [6,7,8], drug delivery [9,10,11], and tissue engineering [12,13,14,15]. Whatever the final destination, materials require a specific grade of mechanical stability that can compete with other properties. In particular, elasticity and swelling efficiencies travel oppositely. Depending on the application, a compromise should be reached; material properties could be opportunely optimized to accomplish a specific application. In light of this, cryogel synthesis plays a key role since it significantly influences the morphology, size, and distribution of the pores, thus tuning the desired properties.

In this view, it is clear that a polymeric cryogel used as support for active metal nanoparticle catalysts in flux bed reactors must possess mechanical properties significantly superior to ones used as a static wound-healing patch [16]. Additionally, cryogels used as scaffolds for cell ingrowth require highly interconnected open porosity to promote tissue neovascularization, and mechanical performances should be carefully balanced. Undoubtedly, the selection of the reactant ratio makes the difference. The literature reports several works on the correlation between the properties of hydrogels and the crosslinker/monomer ratio chosen for the synthesis. Abed et al. (2021) proved that increasing MBAA in hydrogel syntheses decreased swelling parameters. Su et al. (2016) verified the same trend in the swelling behavior of cellulose-based cryogels. Wan et al. (2015) demonstrated the enhancement of the temperature-resistance of hydrogels for higher crosslinker amounts, lowering the space among the grids of the three-dimensional network. As a consequence, an increase in rigidity and temperature resistance was observed [17,18,19]. However, since the morphology and pore connectivity between hydro and cryogels are significantly different, the aforementioned studies cannot be merely transferred to the latter system. On the other hand, the information reported for cryogels in terms of properties/reactant feed relationships is controversial and limited to polyacrylamide, polyacrylic acid sodium salt, and fibroin cryogels [20]. This work aims to extend the scope of these studies and fill the lack of data on HEMA-based cryogel in the literature. To this intention, three cryogels possessing different monomer/crosslinker ratios were synthesized and characterized by morphological (Scanning Electron Microscopy-SEM), thermal (Thermogravimetric Analysis-TGA), and spectroscopical Fourier-transform infrared (FT-IR) points of view. The mechanical properties of the specimens were also evaluated in terms of the compression modulus (Ec) and permanent deformation (εf). Depending on the final cryogel application, an indication of the experimental parameters to prefer was also suggested. Indeed, in the last ten years, HEMA cryogel materials have been largely explored as active support in different applications ranging from environmental and biomedical to energetic purposes [9,21,22]. The main characteristic of HEMA cryogels as adsorbents is that their reactivity is related to the hydroxylic pendant groups that promote H bonds with specific water pollutants, sequestering them [4,23]. Hydroxyl groups can also link semiconductor nanoparticles to the bulk structure for photocatalytic/catalytic applications [24]. Alternately, hydroxyls can be functionalized to anchor hydrophobic groups (e.g., cyclodextrins) for drug release [9]. HEMA cryogels have also been tested to adsorb hydrophilic targets such as albumin, DNA, bilirubin, and lysozyme [25,26,27,28]. As far as it is concerned, we believe that the new data reported in this work can be proficiently used by the readers involved in the aforementioned scientific areas.

## 2. Results and Discussion

### 2.1. Cryogels Synthesis

Table 1 shows the feed ratio and volume reactants used to prepare the HEMA cryogel. The polymerization-averaged yields were also reported. As expected, an increased crosslinker concentration accelerates the formation of networks, resulting in a lower percentage of oligomers or unreacted monomers [29].

### 2.2. Infrared Analysis

**Figure 1:**
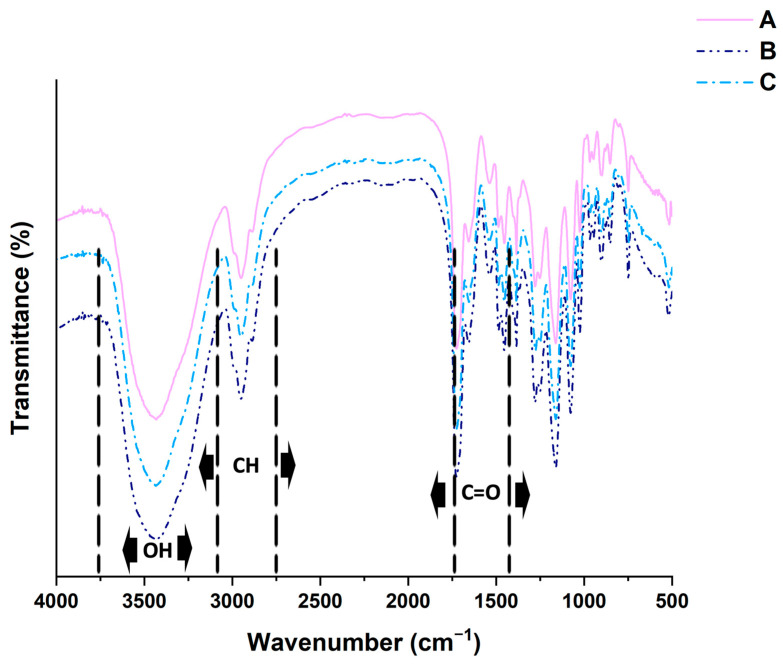
IR-spectra overlay of sample A, HEMA/MBAA 5:1, pink line; sample B, HEMA/MBAA 6:1, dark blue line; and sample C, HEMA/MBAA 6:1, sky blue line.

According to the literature [22], the FT-IR results proved that the reaction was successfully performed for each sample. The main signals identified in the spectra (Figure 1) are the OH stretching at 3443 cm^−1^, the C=O vibration at 1726 cm^−1^ (strong), and the two CH stretching signals at 2929 and 2885 cm^−1^. 

The absence of intense bands at 1640 cm^−1^, related to the C=C double bonds stretching, excludes the presence of considerable amounts of an unreacted monomer, confirming the success of the reactions.

### 2.3. Thermogravimetric Analysis

**Figure 2:**
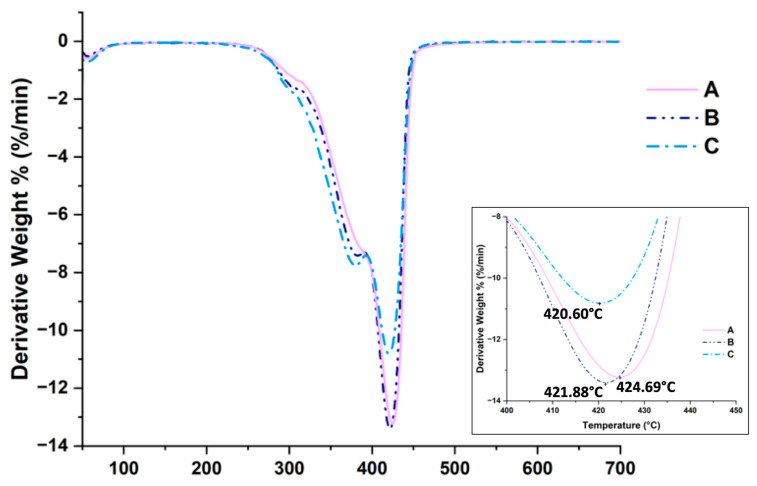
Derivative curves for samples A, B, and C. The inset shows the expansion of the main degradation peak area, where it is possible to appreciate the Tmax for each of the samples.

The TGA gave us information about the thermal stability of samples in an inert condition. The materials’ degradation ranges from 250 °C to 450 °C, with two stages. The first step is characterized by the depolymerization of the material, with HEMA as the main product; the second stage has 2-isopropenyloxy-ethyl methacrylate as the main product [30]. Figure 2 shows the overlay of the derivative curves (DTG) of the three samples; the plots are coherent with the typical thermo-degradation pathway of a HEMA-based polymer [31]. The three temperatures of maximum weight loss of the synthesized samples are pretty close. This is not a surprise since differences are mainly due to the degradation mechanisms concerning the poly-HEMA linkages. The slight differences registered for the samples (Figure 2 and Table 3) are coherent with the ratio between the HEMA/MBAA. Samples A and C have a T_max_ that differs by four °C.

### 2.4. Morphological Analysis

**Figure 3:**
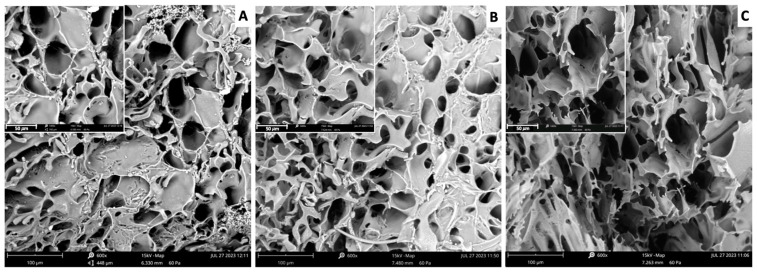
SEM images recorded at 600× zoom of samples (**A**) (HEMA:MBAA 5:1), (**B**) (6:1), and (**C**) (7:1). Insets show the same images at 1400× zoom.

The scanning electron microscopy (SEM) analysis yields valuable information regarding the morphological characteristics of the samples, clearly indicating the presence of highly macroporous materials. The impact of the crosslinker is notably observed in the morphology of the cryogel, and a visible trend emerges as a function of its amount: the number of tiny pores rises, leading to a reduction in overall porosity. Exploiting integrated software, ProSuite 2.9.0.0, within the instrument to compute porosity percentage and average pore diameter, it is possible to reveal that both parameters increase with a higher ratio of crosslinking monomers. Figure 3 shows images of the three specimens at two different zooms (600–1400X). Figure S1 and Table S2 resume the report of the porosimetry software, ProSuite 2.9.0.0, including the main recorded parameters for each sample. As reported for other cryogel systems, the cryo-polymerization process primarily occurs in the unfrozen liquid (*UFLMP*) region, where constant competition exists between cryo-polymerization and ice crystal growth [20]. When the crosslinker concentration is low, ice crystal growth occurs preferentially over cryo-polymerization. This dominance results in the formation of large crystals, developing large pores and thin pore walls. Conversely, smaller pores are formed when the crosslinker concentration is high, accompanied by thicker pore walls [6,32].

### 2.5. Mechanical Analysis

**Figure 4:**
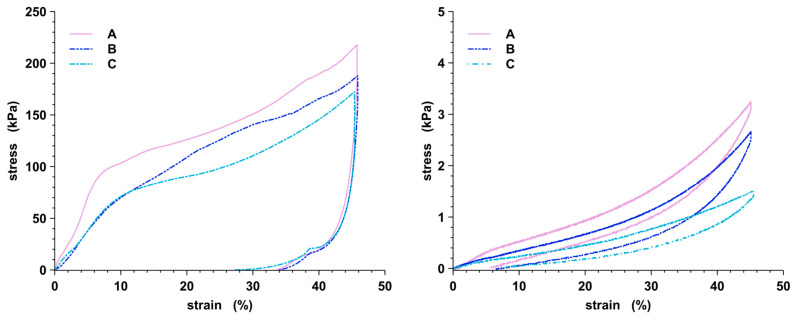
Compression stress–strain curves at a maximum deformation of 45% of dry (**left**) and fully swollen (**right**) cryogels.

Figure 4 shows the compression and tensile curves of the cryogels at the three different crosslinking degrees, averaged on three replicas of the tests both on dry and swollen samples. Concerning the dry ones (Figure 4, left), all the samples exhibit the peculiar behavior of cellular structures, as three different deformation stages can be detected. At minor strain (ε < 10%), the cryogels show a linear elastic deformation, followed by a broad plateau characteristic of the pore structure collapse. Finally, the stress increases again due to material densification. During the tensile test, a large drop in the recorded stress can be noticed, as the cryogels are not able to recover the deformation instantaneously; then, a loose recovery of the initial dimension can be observed over time. At the end of the test, however, the initial dimension is reduced by about 30% for each formulation. The compression modulus (Ec) and the permanent deformation (εf) are reported in Table 2. Ec is normalized to the apparent density (ρ) of the cryogels, calculated as the ratio of the samples’ mass to their apparent volume or the volume of the cylinder characterized by the same diameter and height. As expected, the mechanical properties of the cryogels increase proportionally to the crosslinking degree [33]. In particular, the compression modulus increases with the increase in the crosslinker content, with a more remarkable variation in the 7:1 sample (sample C, light blue line in Figure 4 right). Conversely, the recovery of the deformation is higher for the less-crosslinked sample, as reported in Table 2. Mechanical tests have been performed on wet samples, too (Figure 4, right), to assess their behavior in operating conditions; the three deformation stages are still visible, but when immersed in water, the samples are much softer, and the compression modulus decrease in several orders of magnitude accordingly to the crosslinking degree. However, cryogels can still withstand operating loads. Moreover, the initial dimensions of the cryogels are almost unaltered (εf lower than 8% for all the formulations), as the swollen state contributes to accommodating a fast recovery of the deformations. This is evident by the tensile curves that now closely follow the compressive ones. This peculiar behavior has proven to be fundamental for regeneration purposes.

### 2.6. Swelling 

The ability to incorporate water and swell is an intrinsic characteristic of polymeric gels. The degree of swelling depends on various factors, such as the pH, temperature, and ionic strength of the solution in which the polymer is immersed. In this investigation, our focus was on elucidating the influence of chemical crosslinking variations on the swelling behavior of the polymer.

**Figure 5:**
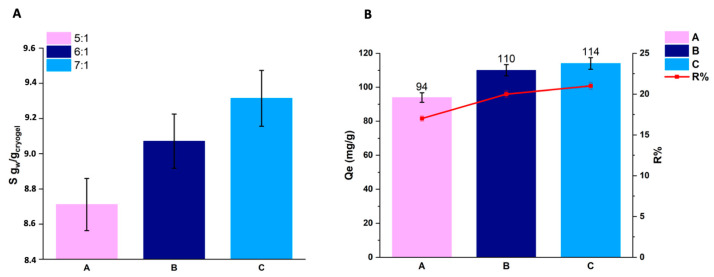
(**A**,**B**) Bars graph on (**A**) swelling degree for samples A, B, and C, and (**B**) Qe (see Equation (2) in Section 4), i.e., the equilibrium absorption capacity. The red line, related to the right *Y*-axis, represents the dye-removal percentage efficiency (see Equation (3) in Section 4).

As delineated in Table 3, all synthesized cryogels can take in significant amounts of water, resulting in a weight increase exceeding 800%. The histograms corresponding to the swelling of each synthesized sample are plotted in Figure 5A. As expected, the lower interconnected crosslinked monolithic specimen (sample C) exhibits a higher water-absorption capacity than sample A, having a higher crosslinked structure. Abed et al. (2021) reported that the heightened presence of crosslinks within the cryogel diminishes the available free volumes between macromolecular chains, making them more susceptible to water penetration. Additionally, another contributing factor that affects swelling could be related to the elevated number of crosslinking bridges that hinder the mobility of macromolecular chains, thus impeding water penetration [17].

### 2.7. Adsorption Test

Cryogels and their properties are extensively investigated for their potential applications in medical and environmental contexts due to their notable capability for capturing and adsorbing/releasing substances. Herein, we examined how the adsorption of methylene violet (MV) at a concentration of 550 ppm was influenced by varying crosslinking rates. As depicted in Figure 5B, all three cryogels exhibit dye adsorption, in line with findings reported by Zagni et al. (2022) [4]. Samples were collected at 5, 10, 15, 30, 60, 180 min, and 24 h. As it turns out, solutions A, B, and C, collected after 24 h, indicate similar values in equilibrium-adsorption capacity (Qe) and percentage-removal efficiency (R%) [34]. In Figure 5B, the red line (R%) and the bars (Qe) show these values corresponding to both measurements. Figure S2 shows the variation of Qt values as a function of time. For all samples, the early 15 min of contact is crucial. In that time frame, samples A, B, and C reached Qe values of 90, 67, and 71, respectively. 

Since adsorption is a relevant property for water remediation and catalytic processes involving pollutant removal [3], information about the trend as a function of network density linked to the resulting mechanical performances is of utmost importance. As it turns out, sample A presents an R% and Qe values that are quite close to sample C. However, their mechanical properties are sensibly different. In this view, the reactant mixture used for sample A should be preferentially selected for these applications. To demonstrate that tiny changes in ingredient ratio can induce a different macroscopical response, we tested the resistance of cryogels under water flux. This measurement can be remarkably informative in the case of cryogel use as a filter. In addition, a scaleup of polymerization was performed, allowing the application of commercial filter holders. For this purpose, we designed a 3D-printed vessel that fitted with the selected commercial filter. The cryo-polymerization of the three samples was performed on this vessel in an industrial fridge. As ready, materials were transferred inside the filter holder and tested by applying a pressure of 0.2 bar with a flux of 500 mL/ min. 

**Figure 6:**
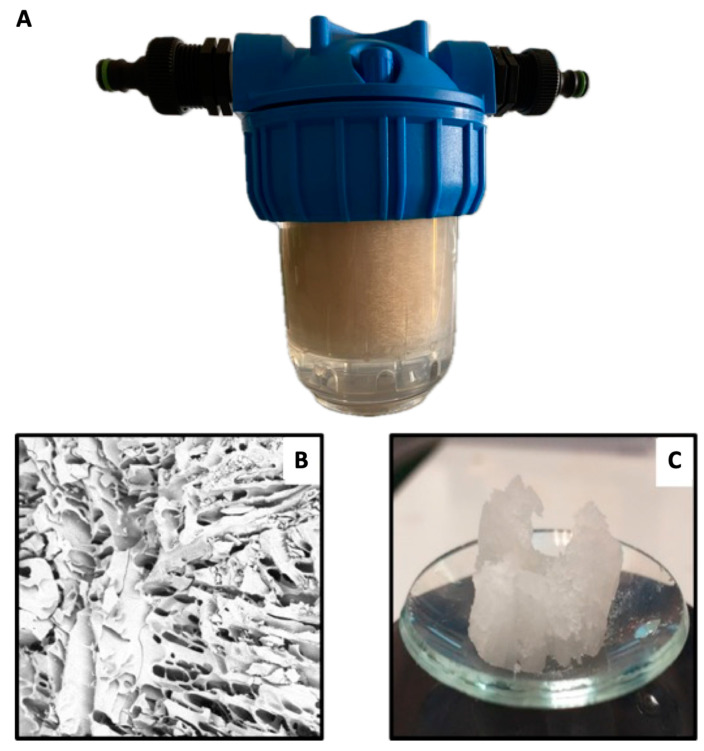
(**A**) Flux test apparatus; (**B**) SEM image of cryopolymer with 20% filler; and (**C**) breakage of 5:1 cryopolymer during swelling tests.

At the end of the test, sample C was visibly damaged. On the contrary, sample A could stand the pressure, exhibiting good material integrity. Figure 6A–C shows the apparatus used for the test, and the SEM image and the photo of the 5:1 sample recorded after the pressure application show that the sample is clearly damaged.

## 3. Conclusions

This study aimed to close a research gap concerning investigations on the chemical and physical characteristics of HEMA cryogels as a function of the amount of crosslinker used in the synthesis. Samples were first characterized through thermal analysis, showing a slightly higher stability in the case of the more crosslinked specimens and a gradually decreasing heat resistance moving toward the less reticulated sample. Based on the final application, the study of the mechanical data made it worth noting the possibility of tuning material performances through control of the HEMA/MBAA ratio. A higher degree of crosslinking can play a key role in obtaining stable cryogels when subjected to a high-pressure flux, repeated use, and regeneration cycles. In this case, a better recovery of the initial deformation (and, therefore, a lower degree of crosslinking) is preferable. Higher crosslinker amounts produced more dense materials with a lower swelling degree. So, the ratio between monomer and crosslinker has to be selected concerning the application of the material. A filtering material constricted in a fixed-dimension filtering system must be produced to avoid excessive swelling and to minimize the resistance to the flux of the treated solution. Fewer pores with larger diameters offer less resistance to the flux, maintaining an elevated superficial area available for the adsorption of the target substances.

## 4. Materials and Methods

### 4.1. Materials

Hydroxyethyl methacrylate (HEMA), N, N’-methylene-bisacrylamide (MBAA), ammonium persulfate (APS), tetra-methyl-ethylene-diamine (TEMED), and methylene violet (MV) were purchased from Sigma-Aldrich and used as received. Deionized water was produced through the Milli-Q water purification system. All chemical structures were made using ACD/ChemSketch 2022.2.0.

### 4.2. Synthesis of Cryogels

Three distinct cryogels (A, B, and C) based on 2-hydroxyethyl methacrylate (HEMA) were synthesized utilizing the radical cryo-polymerization technique. The polymerization involved HEMA/MBAA ratios of 5:1, 6:1, and 7:1. The synthesis process encompassed the preparation of an aqueous solution containing the monomer and crosslinker, with the addition of 1.5% *w*/*w* ammonium persulfate (APS) as a radical initiator and 1.5% *w*/*w* of N,N,N′, N′-tetramethylethylenediamine (TEMED) as a catalyst to facilitate the decomposition of persulfate at low temperatures. After vigorous stirring, the solution was promptly transferred into pre-cooled plastic syringes at 0 °C, obtaining cylindric-shaped monolyte specimens. For each cryogel type, three distinct cryo-polymerization batches were conducted. The procedure continued with the immersion of the syringes in a cryostatic bath at −17 °C for 24 h. Upon achievement of the cryo-polymerization process, the samples were thawed at room temperature and subjected to multiple washes with a mixture of H_2_O/ethyl alcohol/ethyl ether. The concentration of the less volatile solvent was gradually increased during the washing steps, as shown in Table S1. The purified cryogels underwent initial nitrogen purging to eliminate moisture and were subsequently dried using a vacuum pump and a vacuum oven for 24 h [4,5]. Table 1 shows all detailed data on the feed and yield of reactions.

### 4.3. Thermogravimetric Analysis

The thermal stability of the whole set of specimens was verified through a TGA 8000 apparatus (Perkin Elmer Milano–Italy). The analyses were carried out on specimens of about 3 mg under a nitrogen flow (60 mL/min), with a heating ramp of 10 °C/min, in the temperature range from 50 °C to 800 °C. Data were acquired and analyzed through Pyris Software V. 13.3.3.0032, furnished by the producer.

### 4.4. Morphological Analysis

The morphology of each sample was studied through a scanning electron microscopy apparatus (SEM), Phenom Desktop G5, purchased from Phenomenex. Images and data were analyzed through the Phenom-World software ProSuite 2.9.0.0 bundle package, Eindhoven, Netherlands.

### 4.5. Infrared Analysis

The syntheses were monitored by analyzing the samples with a spectrophotometer FT-IR System 2000 (Perkin Elmer Italia, Milan, Italy). The samples were prepared for analysis by mixing the fine powder with KBr previously dried in a vacuum oven overnight at a temperature of 60 °C. The concentration of each sample to KBr was set at 0.5%. Each spectrum is the result of the average of 10 scans at a resolution of 4 cm^−1^.

### 4.6. Mechanical Analysis

Mechanical properties have been evaluated by performing uniaxial compression and tensile tests (Tensometer 2020) both on dry and fully swollen cylindrical-shaped specimens (average diameter, *d*_0_, 8.00 mm, average height, *h*_0_, 12.2 mm). All the samples have been tested by setting 45% of maximum strain at compression and tensile rate of 1 mm min^−1^ and recording the normal force, *F_N_*, and displacement, Δ*h*, over time. Stress (*σ*) and strain (*ε*) have been calculated as *σ* = *F*_*N*_/*A*_0_ (with the A_0_ equivalent cross section calculated on d_0_) and *ε* = Δ*h*/*h*_0_. The compression modulus (Ec) was estimated as the slope of the first linear part of each stress–strain curve. The permanent deformation (εf) has been assessed by measuring the cryogel’s height after the compression and tensile test. Three measurements have been carried out for each sample.

### 4.7. Swelling Test

The ability to uptake water by each sample was verified by conducting a swelling test by weighing the dried specimen and then dipping it in water for two hours. The weight measured after the dipping allows us to calculate the swelling percentage using Equation (1) [19,33,35], where W_swollen_ represents the weight of the sample after solvent uptake, and W_dry_ is the dried weight.

(1) $$S\left(\%\right)=\frac{W_{swollen}-W_{dry}}{W_{dry}}\times 100$$

### 4.8. Adsorption Capacity

Batch tests were conducted to investigate the impact of varying crosslinking amounts on absorption. A solution containing 550 ppm of MV was prepared, and the pH was adjusted to 6.5. To assess the adsorption capabilities of the three differently crosslinked cryogels, 50 mg of each adsorbent was immersed into 50 mL of the MV solution. The mixture was shaken at 150 rpm at 25 ± 0.5 °C for up to 24 h. The residual concentration of the contaminant in the solution was quantified using a UV-Vis V-750 spectrophotometer (Jasco Inc., Easton, MD, USA). Changes in absorption along with the immersion time were measured at the maximum absorption wavelength of MV (595 nm) (Figure S2). The UV calibration curve was reported in supporting information (Figure S3). In evaluating adsorption capacity, two key parameters were calculated: equilibrium-adsorption capacity (Qe) (Equation (2)) and dye-removal percentage efficiency (R%) (Equation (3)) where $C_{0}$ and $C_{e}$ are the concentrations of MV (mg/L) at zero time and after 24 h, V is the solution volume (L), and m is the adsorbent mass (g) [36].

(2) $$Q_{e}=\left(C_{0}-C_{e}\right)\times \frac{V}{m}$$

(3) $$R\%=\frac{\left(C_{o}-C_{e}\right)}{C_{o}}\times 100$$

From our previous investigations (Zagni, EPJ 2022), it has emerged that the model that best represents the adsorption of the dye from pHEMA cryogel is the Langmuir. The latter has been applied to obtain data (Q_e_ and R_L_) on methylene violet. Figure S5 shows the equilibrium adsorption isotherms, and in Table S3, it is possible to find the main parameters obtained by its application to the three cryogels. In light of the values obtained, adsorption is favorable for all three cryogels, with the least crosslinked cryogel, which appears to have the greatest maximum adsorbent capacity (Q_m_) [5].

## Figures and Tables

**Table 1:** Feed ratio, volumes, and yield of the synthesized materials.

Sample	HEMA:MBAA	HEMA (μL)	MBAA (μg)	APS (μL)	TEMED (μL)	Vol H_2_O (μL)	Vol tot (μL)	Y% ^1^
A	5:1	234	59.5	45	45	2633	3000	93
B	6:1	234	49.6	45	45	2640	3000	90
C	7:1	234	42.5	45	45	2645	3000	85

^1^ mass yield averaged over three synthesis batches.

**Table 2:** Mechanical properties of the samples at different crosslinking degrees.

Sample	HEMA: MBAA	ρ [g·cm^−3^] ^3^		Ec [kPa] ^1^	εf [%] ^2^	Ec/ρ [kPa·cm^3^·g^−1^] ^4^
A	5:1	0.145 ± 0.005	dry	17.57 ± 6.31	30.14 ± 3.19	120.19 ± 39.70
			swollen	0.053 ± 0.016	7.090 ± 1.430	0.369 ± 0.107
B	6:1	0.145 ± 0.009	dry	12.15 ± 1.64	32.97 ± 1.31	83.20 ± 6.12
			swollen	0.048 ± 0.006	6.645 ± 0.265	0.331 ± 0.041
C	7:1	0.152 ± 0.012	dry	8.86 ± 2.78	27.22 ± 0.22	57.33 ± 13.74
			swollen	0.036 ± 0.005	5.950 ± 0.860	0.234 ± 0.030

^1^ compression modulus, ^2^ permanent deformation, ^3^ density, and ^4^ compression modulus normalized to the apparent density.

**Table 3:** Summary table of main data of the synthesized materials.

Sample	T_MAX_^1^ (°C)	P % ^2^	D_avg_^3^ (μm)	S% ^4^	Qe ^5^ (mg/g)	R% ^6^	Ec/ρ [kPa·cm^3^·g^−1^] ^7^
A	424 ± 1	30.2	10.8	871.1 ± 2%	94.1	17%	120.19 ± 39.70
B	421 ± 1	34.1	14.5	907.1 ± 2%	109.9	20%	83.20 ± 6.12
C	420 ± 1	46.1	17.4	931.5 ± 2%	113.7	21%	57.33 ± 13.74

^1^ temperature at the maximum rate of weight loss; ^2^ average pore area ratios; ^3^ average pore’s diameter length; ^4^ percentage of swelling; ^5^ equilibrium-adsorption capacity; ^6^ removal percentage efficiency, and ^7^ compression modulus normalized to the apparent density.

## Data Availability

The data presented in this study are openly available in article.

## Supplementary Materials

The following supporting information can be downloaded at: https://www.mdpi.com/article/10.3390/gels10030163/s1, Table S1: Cryogel’s washing parameters; Table S2: Average pore properties calculated on 5 images for each cryogel samples: (A) 1:5; (B) 1:6; (C) 1:7; Figure S1: Cumulative % of pore distribution related to for each cryogel samples: (A) 1:5; (B) 1:6; (C) 1:7 calculated by using Phenom Porometric 1.1.2.0 (Phenom-World BV, Eindhoven, The Netherlands); Figure S2: Variation of MV adsorption values as a function of time of the three synthesized samples; Figure S3: Calibration curve; Figure S4: Thermograms of samples A, B, and C; Table S3: Parameters obtained by applying Langmuir and Freundlich for the three synthesized cryogels; Figure S5: Equilibrium MV adsorption isotherms by plotting Qe versus Ce experimental data for the three synthesized cryogels.
