# Supplementary material for: How the Crosslinker Amount Influences the Final Properties of Hydroxyethyl Methacrylate Cryogels
Source: Gels. 2024 Feb 22;10(3):163. doi: 10.3390/gels10030163 (PMC10969882; doi:10.3390/gels10030163)
Supplement: Supplementary file 1 [file gels-10-00163-s001.zip › gels-2862364-supplementary.pdf]

_bSupporting information

How the crosslinker amount influences the final properties of HEMA Cryogels.

Giuseppe Proietto Salanitri*[1,2], Enrica Luzzi [3], Daniele Caretti[2], Tommaso Mecca[4], Sabrina C. Carroccio[1], and Andrea A. Scamporrino[1]

[1] CNR-Institute for Polymers, Composites and Biomaterials, Via Paolo Gaifami 18, 95126 Catania, Italy; sabrinacarola.carroccio@cnr.it (S.C.C.); andreaantonio.scamporrino@cnr.it (A.A.S.)
[2] Department of Industrial Chemistry "Toso Montanari", University of Bologna, Viale Risorgimento 4, 40136 Bologna, Italy; daniele.caretti@unibo.it
[3] Department of Chemical Engineering, Materials and Industrial Production, University of Naples Federico II, DICMaPI–P. le Tecchio 80, 80125 Naples, Italy; enrica.luzzi@unina.it
[4] CNR-ICB, Via Paolo Gaifami 18, 95126 Catania, Italy; tommaso.mecca@icb.cnr.it
* Correspondence: giuseppe.proietto3@unibo.it

Table S1. Cryogel's washing parameters

CYCLE N°	WASHING SOLUTION	METHOD
1st	33 mL H_2O	Directed percolation
2nd	33 mL H_2O-EtOH 50:50	Directed percolation
3rd – 4th	H_2O-EtOH 25:75	Immersion
5th - 6th - 7th	EtOH	Immersion
8th	EtOH- Et_2O 50:50	Immersion
9th – 10th – 11th	Et_2O	Immersion

Table S2. Average pore properties calculated on 5 images for each cryogel samples: (A) 1:5; (B) 1:6; (C) 1:7.

SAMPLE	A		B		C	
Pore Tot. Number	393		238		168	
Av Pore Area Ratio	39,34%		31,10%		46,12	
	**Median**	**Average**	**Median**	**Average**	**Median**	**Average**
Circle eq. diameter	9.09 µm	12.8 µm	11.8 µm	14.5 µm	12.3 µm	17.4 µm
Major axis	12.3 µm	18.4 µm	16.4 µm	20.7 µm	18.7 µm	25.5 µm
Minor axis	6.53 µm	9.36 µm	8.01 µm	10.7 µm	8.49 µm	12.7 µm
Circumference	31.7 µm	48.4 µm	41.1 µm	54.2 µm	46.4 µm	73.9 µm
Convex hull	31.7 µm	45.2 µm	39.8 µm	50.8 µm	45.4 µm	66.1 µm
Circum. circle diam.	12.7 µm	18.6 µm	16.2 µm	20.9 µm	18.9 µm	27.2 µm
Area	64.9 $µm^2$	200 $µm^2$	110 $µm^2$	238 $µm^2$	118 $µm^2$	406 $µm^2$
Volume by area	394 $µm^3$	3.82E+03 $µm^3$	868 $µm^3$	4.44E+03 $µm^3$	965 $µm^3$	1.21E+04 $µm^3$
Pixel count	340	1049	576	1247	618	2126
Aspect ratio	0.581	0.573	0.588	0.574	0.57	0.561
Circularity	0.798	0.777	0.814	0.77	0.742	0.719
Convexity	1	0.974	1	0.972	0.989	0.956
Elongation	0.419	0.427	0.412	0.426	0.43	0.439
Grayscale	183	183	175	175	187	187
Inscrib. circle diam.	6.21 µm	9.02 µm	8.18 µm	9.94 µm	8.07 µm	11.3 µm

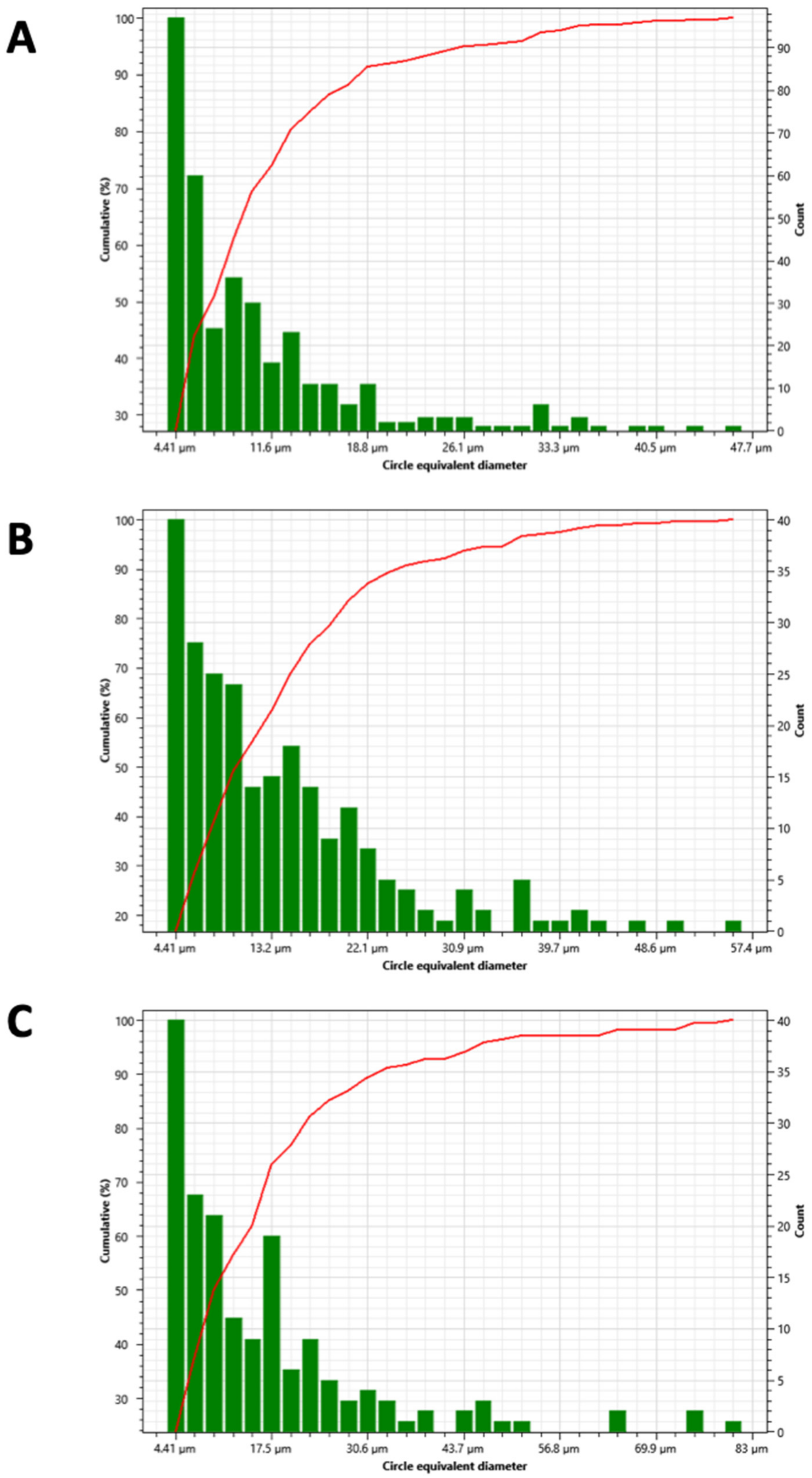

Figure S1. Cumulative % of pore distribution related to for each cryogel samples: (A) 1:5; (B) 1:6; (C) 1:7 calculated by using Phenom Porometric 1.1.2.0 (Phenom-World BV, Eindhoven, The Netherlands).

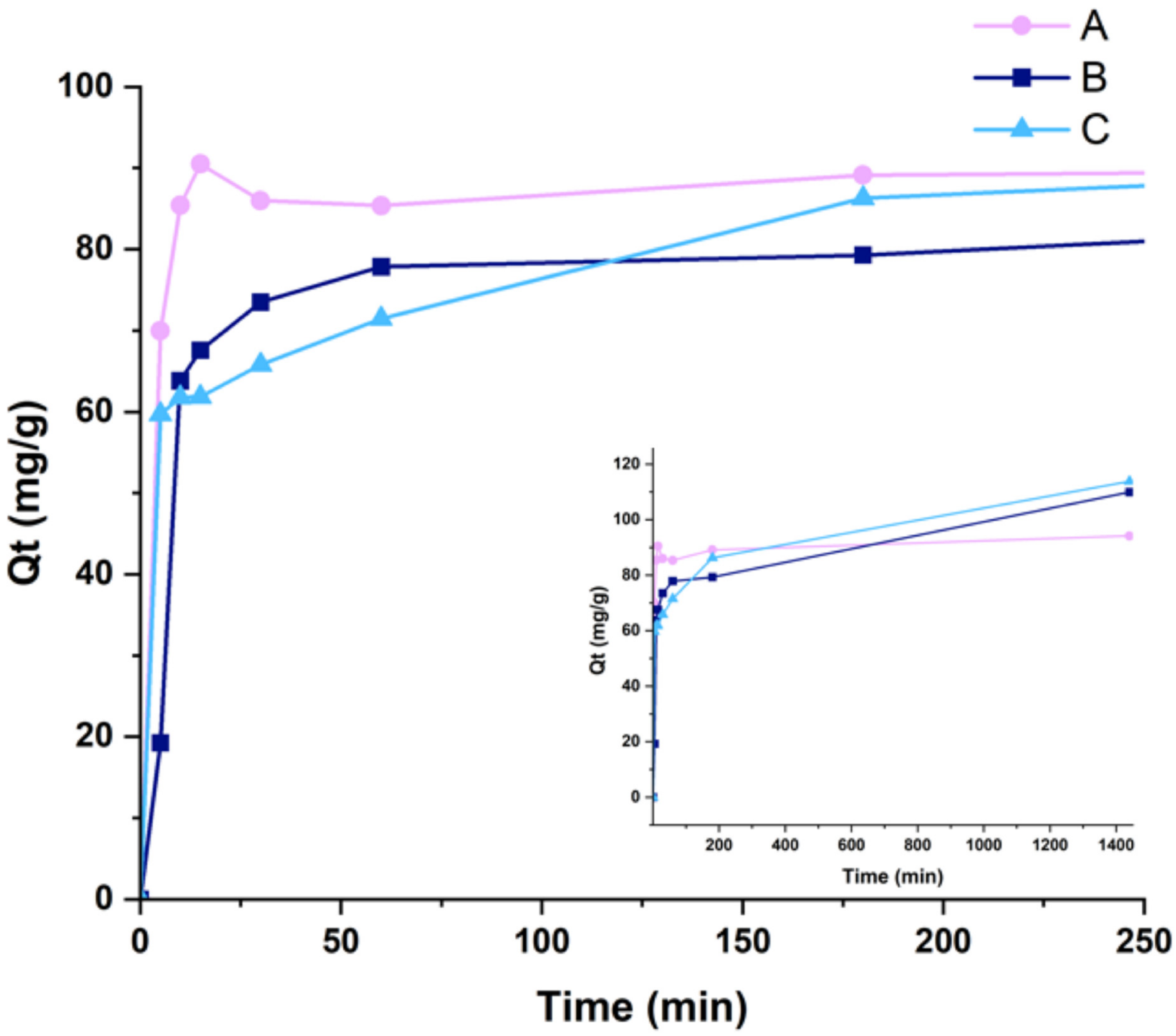

Figure S2. Variation of MV adsorption values as a function of time of the three synthesized samples.

Equation	Y = a + b * x
Plot	C
Weight	No Weighting
Intercept	-7,03168E -4 ± 0,004
Slope	0,12849 ± 6,45411E
Residual Sum of Squares	2,72323E-5
Pearson's r	0,99997
R-Square (COD)	0,99995
Adj. R-Square	0,99992

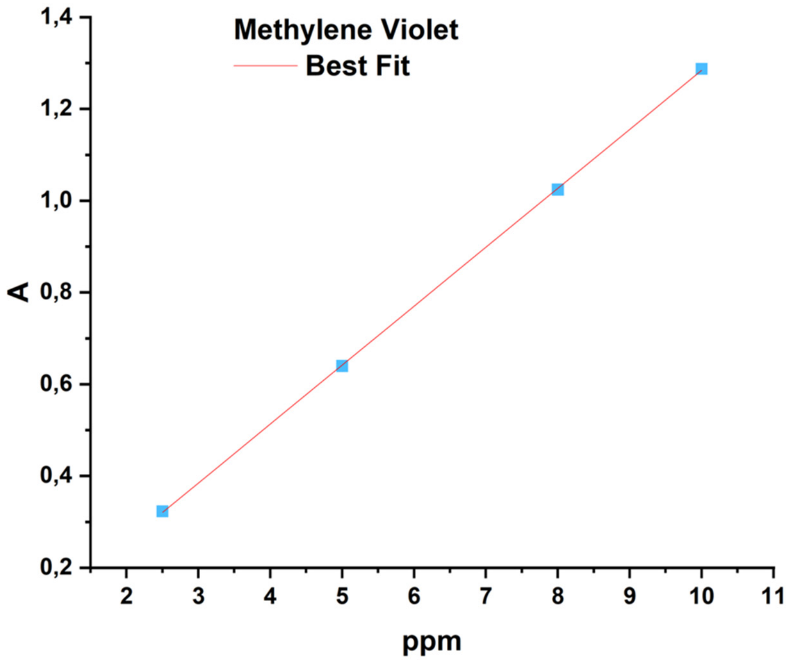

Figure S3. Calibration curve

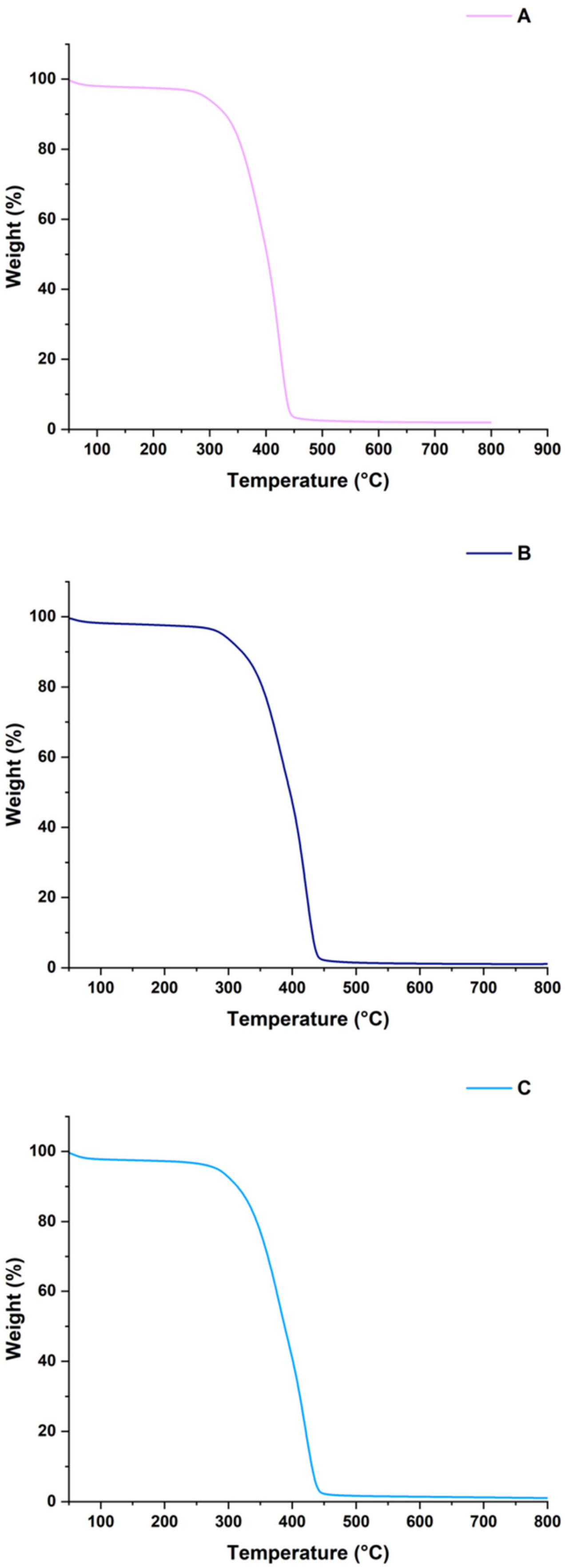

Figure S4. Thermograms of samples A, B, and C.

Table S3. Parameters obtained by applying Langmuir and Freundlich for the three synthesized cryogels.

	Langmuir Model Parameters			
	*Q_m (mg/g)	K_L	R_L	R2
A	94	20,2	0.00009	0.9624
B	110	0.0308	0.0556	0.9529
C	114	0.0327	0.0494	0.9616

* Maximum adsorption capacity

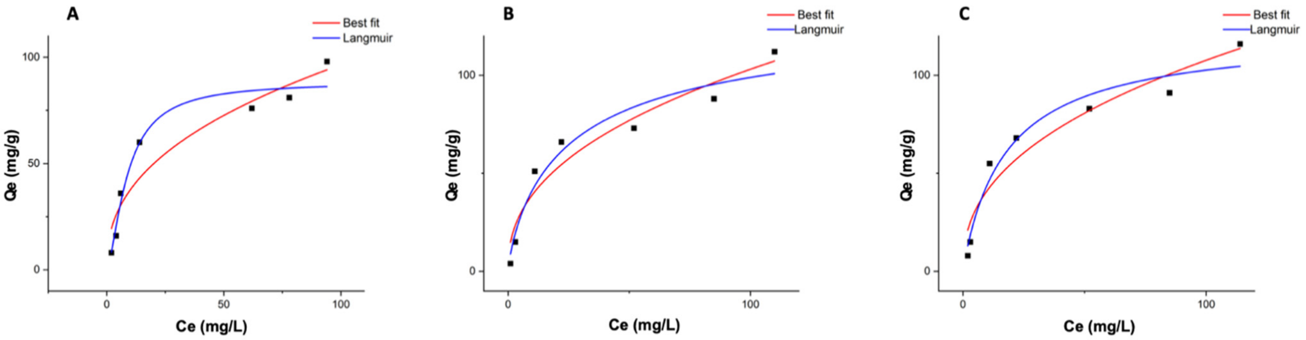

Figure S5. Equilibrium MV adsorption isotherms by plotting Qe versus Ce experimental data for the three synthesized cryogels.

Langmuir model is described by Equation S1, where **qe** (expressed in mg/g) represents the amount of methylene violet adsorbed, **Ce** (mg/L) is the concentration at the equilibrium, $\mathbf{Q_0}$ (mg/g) is the monolayer capacity, and **b** (mg/L) represents a constant whose value is related to the heat of adsorption ($\mathbf{K_L}$).

Eq. S1: $$q_e = \frac{Q_0 b C_e}{1+bC_e}$$

The following Equation S2, may be used to determine the separation factor ($\mathbf{R_L}$), related to the adsorption process' efficiency, from $\mathbf{K_L}$ value

Eq. S2: $$R_L = \frac{1}{1+K_L C_0}$$
